# Identifying targets for interventions to support public adherence to government instructions to reduce transmission of SARS-CoV-2

**DOI:** 10.1186/s12889-021-10574-6

**Published:** 2021-03-17

**Authors:** Christopher J. Armitage, Chris Keyworth, Jessica Z. Leather, Lucie Byrne-Davis, Tracy Epton

**Affiliations:** 1 Manchester Centre for Health Psychology, School of Health Sciences, University of Manchester, Manchester, M13 9PL UK; 2 Manchester University NHS Foundation Trust, Manchester Academic Health Science Centre, Manchester, M13 9PL UK; 3 NIHR Greater Manchester Patient Safety Translational Research Centre, University of Manchester, Manchester, M13 9PL UK; 4 Division of Medical Education, School of Medical Sciences, University of Manchester, Manchester, M13 9PL UK

**Keywords:** COVID-19, Adherence, Intervention, SARS-CoV-2, Hand hygiene, Physical distancing, COM-B

## Abstract

**Background:**

SARS-CoV-2 lacks sentience and can only be spread through human behaviour. Government instructions to the general public include: (a) limiting time spent outside the home, (b) staying more than 1 m away from people outside the household at all times, and (c) maintaining hand hygiene. Current evidence suggests high rates of adherence to such instructions, but interventions to sustain adherence to government instructions in the long term can only be developed if we know why people do or do not adhere to them. The aims were to assess levels of public adherence to government instructions to reduce transmission of SARS-CoV-2, but more importantly to gauge why people were or were not adhering to instructions.

**Methods:**

Cross-sectional survey of 2252 adults who were representative of the UK population. Data were analysed descriptively, and using one-sample *t*-tests, within-participants ANOVA and multiple linear regression.

**Results:**

The sample reported mostly adhering to UK government instructions to reduce SARS-CoV-2 transmission, with 5% or fewer people reporting active resistance to instructions. People generally reported high levels of capability, opportunity and motivation to follow the instructions, but perceived relatively few physical and social opportunities. Multiple linear regression analyses showed that better adherence was associated with older age, being a woman, having a white ethnic background, and with perceiving greater levels of capabilities, opportunities and motivations.

**Conclusions:**

Interventions targeted at people with black, Asian and minority ethnic backgrounds, men and younger people that focus on increasing capabilities, providing greater opportunities and boosting motivations are needed to support continued adherence to government instructions to reduce SARS-CoV-2 transmission. Further research is required to track changes in people’s capabilities, opportunities, motivations and behaviours in response to the ongoing emergency, any changes in government instructions, and to adapt the present procedures to other emergency situations.

## Background

As of 18 January 2021, the severe acute respiratory syndrome coronavirus-2 (SARS-CoV-2) has caused in excess of 2,000,000 deaths from coronavirus disease 2019 (COVID-19) [1]. Considerable resource has rightly been allocated to understanding the biological underpinnings to COVID-19 with a view to developing screening tools and interventions to tackle the pandemic [2]. However, the psychosocial underpinnings to COVID-19 are equally important because the public plays an important role in preventing the spread of the virus by: (a) limiting the time spent outside; (b) staying more than 1 m away from people outside the household at all times; and (c) maintaining hand hygiene [3]. The evidence suggests that, at present, public adherence to the instruction to stay at home is above 80% [4], but it is less clear whether instructions to distance physically and maintain hand hygiene are being followed. Moreover, as periods of lockdown extends and the likelihood is that some measures may remain in place for years [5], it is important that adherence is sustained. The only way to ensure continued voluntary adherence is first to understand why people are, or are not, adhering to government COVID-19-related instructions to stay at home, keep physically distant and maintain hand hygiene to prevent spread of the SARS-CoV-2 virus [3]. The broad aim of the present research was to gather data to inform behaviour change interventions designed to promote sustained adherence to government COVID-19-related instructions. The specific aims were to: assess levels of adherence to government COVID-19-related instructions to stay at home, keep physical distance and maintain hand hygiene among the general public; gauge people’s perceptions of their capabilities, opportunities and motivations [6]; and identify predictors of adherence.

Mortality rates associated with COVID-19 among men, people with black, Asian and minority ethnic backgrounds and/or people with low socioeconomic status have been higher than in the broader population [7]. Thus, although adherence to UK government COVID-19-related instructions is high at a population level, it is plausible that adherence is more problematic for people already suffering inequalities. Understanding which groups to target with interventions will be important in developing strategies to ensure sustained adherence to government instructions. Evidence from the United States suggests that older people may be more likely to distance physically [8]; in the Democratic Republic of the Congo non-adherence was associated with being unemployed and being a woman [9], whereas women were more likely to adhere in Hong Kong [10], meaning there may be cultural differences in patterns of adherence.

The present study aims to assess the level of adherence to government COVID-19-related instructions in a large sample that is representative of the UK population, and for the first time to understand why people are or are not adhering. It is hypothesized that people in general will: (a) be largely adherent to government COVID-19-related instructions; (b) feel capable, motivated with sufficient opportunities to adhere to government COVID-19-related instructions; but that (c) people from vulnerable groups, including those from black, Asian and minority ethnic groups and those with low socioeconomic status find it harder to adhere to government COVID-19-related instructions.

## Method

### Study design and participants

The study design was cross-sectional.

### Sampling

YouGov, a market research company, recruited a sample of 2252 UK residents aged 18+ from their existing database, as part of their daily “omnibus” survey that includes questionnaire items designed to assess public opinions and are not reported here. A sample of adults designed to be representative of the UK population was invited to take part in an online questionnaire and were incentivised in line with YouGov’s points system [11, 12]. The data were sent securely to the research team for analysis. Ethical approval was obtained from a University Research Ethics Committee (ref: 2020–9551-15,105) and participants gave written informed consent at the beginning of the survey. It was not appropriate or possible to involve patients or the public in the design, or conduct, or reporting, or dissemination plans of our research.

### Instrument

#### Sociodemographic variables

Sociodemographic measures of age, gender, ethnicity and socioeconomic status were taken using standard UK Office for National Statistics [13] measures.

#### Psychosocial variables

Keyworth et al.’s [14] measure was used to assess people’s capabilities, opportunities and motivations with respect to adhering to UK government COVID-19-related instructions. The measure is based on Michie et al.’s (6] capabilities, opportunities and motivations model, which is designed to capture all the key drivers of human behaviour. The UK National Institute for Health and Care Excellence endorses the model as a key theoretical framework for understanding and supporting behaviour change [15]. Keyworth et al.’s [14] measure comprises six items designed to tap physical capability, psychological capability, physical opportunity, social opportunity, reflective motivation, and automatic motivation, which are presented in Table 1. The items are accompanied by brief definitions of each of the constructs (e.g., the reflective motivation item is accompanied with: “What is motivation? Conscious planning and evaluation (beliefs about what is good and bad) (e.g., I have the desire to, I feel the need to) and are assess on 11-point scales (*strongly disagree*[0]-*strongly agree*[10].

**Table 1:** Sociodemographic, psychosocial and behavioural characteristics of the sample

Variable	%	*M*	*SD*
Gender			
Men, *n* = 1092	48.5	–	–
Women, *n* = 1160	51.5	–	–
Age	–	48.26	17.50
Social Grade			
Non-manual, *n* = 1284	57.0	–	–
Manual / unemployed, *n* = 968	43.0	–	–
Ethnicity			
White, *n* = 2095	93.0	–	–
Black, Asian, Minority Ethnic/Prefer not to say, *n* = 157	7.0	–	–
Overall Adherence (0–10)	–	8.84	1.54
Resistance to Staying Inside	5.0	–	–
Resistance to Physical Distancing	4.3	–	–
Resistance to Hand Hygiene	3.0	–	–
Physical Capability: “I am PHYSICALLY able to follow the UK government’s COVID-19-related instructions” (0–10)		7.72	2.34
Psychological Capability: “I am PSYCHOLOGICALLY able to follow the UK government’s COVID-19-related instructions” (0–10)		7.55	2.30
Physical Opportunity: “I have the PHYSICAL opportunity to follow the UK government’s COVID-19-related instructions” (0–10)		7.37	2.45
Social Opportunity: “I have the SOCIAL opportunity to follow the UK government’s COVID-19-related instructions” (0–10)		7.13	2.55
Reflective Motivation: “I am motivated to follow the UK government’s COVID-19-related instructions” (0–10)		7.71	2.40
Automatic Motivation: “Following the UK government’s COVID-19-related instructions is something that I do automatically” (0–10)		6.83	2.45

### Adherence

Participants rated their adherence to government COVID-19-related instructions on an 11-point scale (*not at all*[0]-*very much so*[10]) using the item, “How closely are you following the UK government’s COVID-19-related instructions?”. Participants were also asked to describe how they were following government COVID-19-related instructions using an open-text question.

### Data collection

The data were collected via an online survey on 30th April 2020.

### Operational definition of variables

Consistent with the ethnic profile of the UK, ethnicity was divided into White versus Black, Asian or Minority Ethnic and socioeconomic status was divided into manual versus non-manual. Responses to the open-ended text question were coded according to how participants were adhering to the three UK government instructions that were in place at the time of data collection: (1) only going outside for food, health reasons (including exercise) or work (but only if cannot work from home (including self-isolation)); (2) social distancing (if going out, stay 2 m away from other people at all times; and (3) washing hands for at least 20 s or more often than usual. Responses were double coded by three members of the research team (JZL, TE, and CK) for resistance (explicitly reported not doing), reported doing, or not mentioned. Cohen’s kappa between coders showed excellent agreement for staying at home (κ = 0.86), social distancing (κ = 0.91), and hand washing (κ = 0.95). Any disagreements were discussed and agreed upon, with the use of an independent third coder where necessary.

### Statistical analyses

Data were weighted to ensure analyses properly reflected the UK population. Descriptive statistics were used to characterize the population and to illustrate levels of adherence, and perceptions of capabilities, opportunities and motivations. One-sample *t*-tests, within-participants ANOVA and deviation contrasts were used to explore differences between levels of capabilities, opportunities and motivations with respect to adhering to instructions. Adherence was entered as a dependent variable in multiple linear regressions to examine associations between sociodemographic factors, psychosocial variables, and adherence. Because the psychosocial variables were intercorrelated (*r*s > .45) separate linear regression models were used for each capability, opportunity and motivation variable, and each model was adjusted for potential correlates of adherence (age, gender, ethnicity, and social grade).

## Results

### Participant characteristics

Consistent with the sampling frame, the sample was broadly representative of the UK population [13]. As presented in Table 1, most participants were white (93.0%) and half were women (51.5%) and roughly evenly split between people in non-manual (57.0%) and manual occupations / unemployed (43.0%). Mean age was 48.26 years (*SD* = 17.50). Consistent with opinion poll data, adherence with instructions was reportedly high (*M* = 8.84 on a 0–10 scale) and there was very little evidence of resistance (<=5.0%) to instructions to stay at home, keep physical distance and maintain hand hygiene. Participants reported being capable, motivated and having opportunities to follow government instructions with scores significantly above the midpoints of the 0–10 scales (*t*s > 39.50, *p*s < .01; Table 1).

Respondents scored highest in terms of their physical capability (*M* = 7.72 on a 0–10 scale) and reflective motivation (*M* = 7.71 on a 0–10 scale), meaning that they felt they had the necessary skills and stamina, and were consciously and actively wanting to follow government instructions. However, they reported significantly lower: automatic motivation (e.g., habit; 95%CI versus physical capability = − 1.03, − 0.75; versus reflective motivation = − 1.01, − 0.74), psychological capability (e.g., knowledge; 95%CI versus physical capability = − 0.27, − 0.06; versus reflective motivation = − 0.27, − 0.03) perceived fewer physical opportunities (e.g., necessary resources; 95%CI versus physical capability = − 0.46, − 0.22; versus reflective motivation = − 0.45, − 0.19) and social opportunities (e.g., other people following instructions; 95%CI versus physical capability = − 0.73, − 0.47; versus reflective motivation = − 0.71, − 0.44).

### Associations between Sociodemographic variables, psychosocial variables and adherence

Multiple linear regression (Table 2) showed that women, older people and people with a white ethnic background were most likely to report adhering overall to government instructions. Controlling for these sociodemographic variables, people’s perceptions of their capabilities, opportunities and motivations were also significantly associated with adherence to government instructions.

**Table 2:** Associations between sociodemographic variables, psychosocial variables and adherence

Variable	*B*	*SE*	*95% CI*	*p*
Gender	0.46	0.06	0.34, 0.59	< 0.01
Age	0.01	0.01	0.01, 0.02	< 0.01
Social Grade	−0.01	0.06	−0.14, 0.11	0.83
Ethnicity	−0.29	0.12	−0.53, − 0.05	0.02
Physical Capability	0.06	0.02	0.02, 0.09	< 0.01
Psychological Capability	0.13	0.02	0.09, 0.17	< 0.01
Physical Opportunity	0.08	0.02	0.05, 0.11	< 0.01
Social Opportunity	0.08	0.02	0.05, 0.12	< 0.01
Reflective Motivation	0.18	0.02	0.15, 0.21	< 0.01
Automatic Motivation	0.07	0.02	0.04, 0.09	< 0.01

## Discussion

### Principal findings

Adherence to government instructions was generally high, particularly among older people, women and people with a white ethnic background, and there was little evidence of resistance to instructions. People’s perceptions of their capabilities, opportunities and motivations were high, although it should not be taken for granted that this will remain the case.

### Strengths and limitations

Although the sample was large and representative, the cross-sectional design means that causality cannot be inferred. Also, it was not possible to obtain objective measures of whether or not individuals stayed at home, stayed 2 m or more (as per UK government instructions at the time) from people who were not members of the household, and hand hygiene.

### Previous studies

Consistent with data collected by the UK Office for National Statistics, we found high levels of adherence to government instructions. The findings that men and people with black, Asian and minority ethnic backgrounds were less likely to be adherent are consistent with higher mortality rates in these populations [7]. Preliminary UK data also suggest that younger people are less adherent [16], which is consistent with the present findings. Globally, the evidence suggests that age and socioeconomic status may be more closely associated with adherence than in the UK [8, 9], meaning there may be cultural differences in patterns of adherence that would need to accounted for in public health interventions. We also found further evidence for the predictive validity of the capabilities, opportunities and motivations model of behaviour change [6, 14]. As far as we are aware, the present study is only the second to have tested the predictive validity of the Keyworth et al. [14] measure. This is important because it provides the basis for interventions to sustain and improve adherence to government instructions. For example, people perceived relatively few social opportunities, so it would be possible to explore legislative, social planning and/or service provision options to enable people to adhere to the instructions.

### Implications

Sustaining people’s capabilities, opportunities and motivations will be key to promoting continued adherence to government instructions. Developing interventions to boost capabilities, opportunities and motivations to adhere to government instructions among younger people, men and people with black and Asian minority backgrounds will be particularly important. Automatic motivation was particularly low meaning that interventions to establish new habits and help people to regulate their emotions may be particularly effective at changing and sustaining people’s adherence behaviours [17].

### Future research

Further work is required to track changes in adherence to government instructions, particularly as they evolve during the course of the COVID-19 pandemic. It would also be valuable to act now to understand and anticipate the future changes in behaviour that government may wish the public to make, such as adhering to instructions to self-isolate, attending screening, adherence to medications and take up of vaccinations. It may be possible in future research to harness the power of new technology to track precisely the position and physical distancing of individuals and develop sensors that could measure hand hygiene, but this would raise serious ethical questions and may not be desirable.

## Conclusions

Although government instructions are changing rapidly, the present research is a first step in understanding why people are adhering to instructions and what steps may need to be taken to ensure continued adherence. Interventions that target younger people, men and people with black, Asian and minority ethnic backgrounds that explicitly promote habit formation and provide people with physical and social opportunities are most likely to promote adherence.

## Data Availability

The dataset used for the current study is available via the Open Science Framework https://osf.io/tkhuf.
